# Correction to: Efficacy of single-site radiotherapy plus PD-1 inhibitors vs PD-1 inhibitors for oligometastatic non-small cell lung cancer

**DOI:** 10.1007/s00432-021-03877-z

**Published:** 2021-12-08

**Authors:** Peiliang Wang, Tianwen Yin, Kaikai Zhao, Jinming Yu, Feifei Teng

**Affiliations:** 1 Department of Radiation Oncology, Shandong Cancer Hospital and Institute, Cheello College of Medicine, Shandong University, Jiyan Road 440, Jinan, 250117 Shandong Province People’s Republic of China; 2 Department of Radiation Oncology, Yantai Afliated Hospital of Binzhou Medical University, Yantai, Shandong People’s Republic of China; 3 Department of Radiation Oncology, Shandong Cancer Hospital and Institute, Shandong First Medical University, Shandong Academy of Medical Sciences, Jinan, Shandong People’s Republic of China

## Correction to: Journal of Cancer Research and Clinical Oncology 10.1007/s00432-021-03849-3

In the original article published, the authors Peiliang Wang and Tianwen Yin are co-first authors and have equally contributed to this work.

The original article has been corrected.

